# Region-specific proteomic profiling of brain interstitial fluid *via* a micro-invasive sampling platform

**DOI:** 10.1039/d6lc00038j

**Published:** 2026-04-13

**Authors:** Qun Cao, Hannah D. Jackson, Aidan J. Duncan, Yufei Cui, Haley O. Higginbotham, Stella Lesnik, Yunseo Jo, Jiaquan Yu, Forest M. White, Michael J. Cima

**Affiliations:** a Koch Institute for Integrative Cancer Research, Massachusetts Institute of Technology Cambridge MA USA mjcima@mit.edu; b Harvard-MIT Program in Health Sciences and Technology, Massachusetts Institute of Technology Cambridge MA USA; c Department of Biological Engineering, Massachusetts Institute of Technology Cambridge MA USA; d Department of Mechanical Engineering, Massachusetts Institute of Technology Cambridge MA USA; e Department of Materials Science and Engineering, Massachusetts Institute of Technology Cambridge MA USA

## Abstract

Interstitial fluid (ISF) within the brain parenchyma contains secreted factors related to brain function, metabolism, and neurodegenerative disorders. Cerebrospinal fluid (CSF) is commonly sampled due to its accessibility in well-defined spaces, but it does not fully capture the diversity of the brain secreted proteome. The brain is remarkably heterogeneous on a millimeter scale or smaller, and the secreted proteome likely reflects that heterogeneity. Traditional methods like cerebral microdialysis suffer from recovery loss and tissue damage due to semipermeable membranes and large probe sizes. This study develops a micro-invasive, membrane-free platform for ISF sampling, enabling mass spectrometry analysis of small sample volumes with high spatial resolution and minimal tissue damage. Also, this platform collects samples within approximately 15 minutes, representing a major reduction in sampling time compared to microdialysis. We analyzed proteomic profiles of ISF from the nucleus accumbens and substantia nigra, revealing significant differences in protein abundance and composition indicative of their biological functions. We also compared ISF with CSF and found significantly more proteins associated with brain-specific functions, such as synaptic transmission and vesicle-mediated transport. This novel ISF sampling method can enhance clinical liquid biopsy techniques for brain diseases and provides insights into distinct molecular profiles of different brain regions.

## Abstract 2

This study presents a localized, membrane-free method for sampling brain interstitial fluid, enabling high protein yield from small volumes. It uncovers proteomic differences across brain regions, supporting improved biomarker detection.

## Graphical abstract

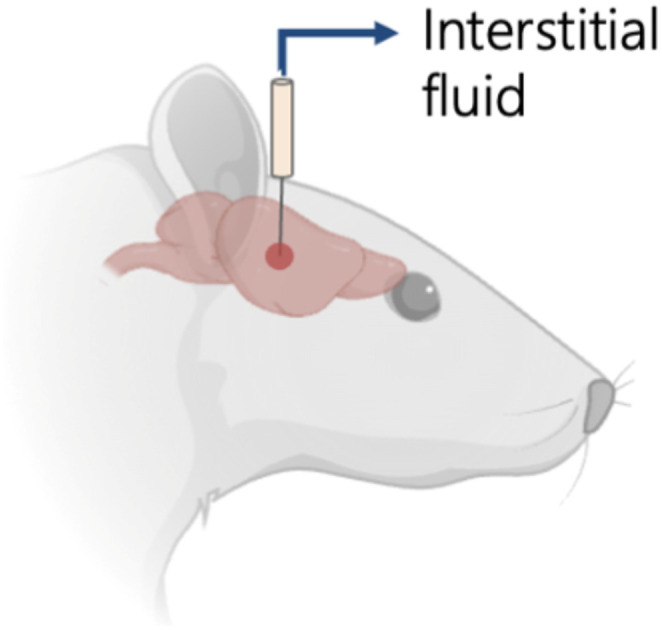

## Introduction

The composition of the brain microenvironment offers clues to brain function and metabolism.^1–6^ The analysis of brain interstitial fluid enables biomarker discovery studies and facilitates the diagnosis and knowledge of various neurodegenerative central nervous system disorders.^7–11^ Brain cells are supported and protected by two major fluids: interstitial fluid (ISF) and cerebrospinal fluid (CSF). ISF occupies the interstitial spaces between the cellular compartments of the brain, while CSF fills the cerebral ventricles and the subarachnoid space.^12^ CSF is frequently collected in liquid biopsies because it occupies a large volume in well-defined spaces such as the ventricles and subarachnoid space.^13–15^ Sampling CSF is well established in clinical practice, typically performed *via* lumbar puncture in the subarachnoid space,^16,17^ or less frequently, through suboccipital puncture from the cisterna magna.^18–20^ Sampling ISF is more challenging than CSF as recovery of fluid volume is constrained by the tight spaces between neurons and glial cells.^21,22^ Collecting ISF also requires potentially more invasive procedures that could disrupt brain tissue and cellular structures.^23,24^ Both ISF and CSF contribute to the dynamic environment that supports neuronal function and metabolic processes, but ISF surrounds the parenchymal cells of the brain and provides a direct medium for the supply of nutrients, removal of waste, and intercellular communication.^25–28^ Neurochemicals that are actively transported in the ISF are likely much less concentrated in the CSF.^21^ These neurochemicals are particularly important because they may provide the most insight into brain function and dysfunction. ISF sampling is therefore critical for understanding neural network dynamics in both physiological and pathological states.

The most common method for ISF sampling is cerebral microdialysis.^29–34^ This technique uses a semi-permeable membrane over the end of a multi-lumen probe allowing substances in the extracellular space to move according to their concentration gradient. The flow of fluid within the probe maintains this gradient and facilitates the transport of the sample out of the brain for analysis.^35^ Microdialysis enables continuous sampling of ISF and is versatile in detecting a broad range of analytes, including small molecules like neurotransmitters and metabolites, and sometimes larger molecules such as peptides and proteins.^36,37^ Traditional microdialysis is limited by its semi-permeable membrane, however, which causes recovery loss and prevents the measurement of dense core extracellular vesicles which are important for cell signaling. The typical large size of microdialysis probes also limit spatial resolution and cause significant damage to brain tissue, making the technique unsuitable for longitudinal sampling.^33,38,39^ Implanted microelectrodes are also used to monitor real-time dynamics of neurochemicals, but electrochemical methods are limited to detecting only redox-active analytes and do not fully represent the ISF components.^40–47^ Membrane-free open-flow approaches such as cerebral open flow microperfusion (cOFM) also enable continuous ISF sampling and have been used mainly for brain PK/BBB studies, with emerging reports extending to larger molecules such as antibodies.^34,48–50^ Membrane-free ISF sampling using push–pull perfusion (PPP) has been demonstrated and refined extensively by Kennedy and others, including early low-flow/microfluidic PPP implementations,^51–53^ segmented-flow PPP for high temporal and spatial resolution,^54–56^ and microfabricated sampling probes integrated with droplet-based microfluidics and mass spectrometry.^57,58^ Tissue impact under low-flow PPP conditions has also been evaluated.^59^ Related microfabricated probe architectures include droplet-based microdialysis collection systems and *in vivo* droplet-based monitoring,^60–62^ and miniaturized PPP probes for few-second neurotransmitter sampling.^63,64^ Advances in microfabricated membrane microdialysis probes and nanodialysis platforms also improved sampling performance.^65,66^ These prior studies demonstrate the feasibility of membrane-free and microfabricated perfusion sampling; however, most were designed for neurochemical monitoring^58,67,68^ and do not explicitly address the challenges of low-loss proteomic sampling from sub-microliter volumes, where dead volume and surface adsorption can strongly influence recovery. There remains a need for micro-invasive and membrane-free ISF sampling methods which are compatible with comprehensive proteomic profiling while minimizing tissue disruption, dead volume, and adsorption losses.^69–71^

Our lab previously developed a micro-invasive probe for brain ISF sampling and demonstrated its proof of concept for detecting extracellular proteins using mass spectrometry.^23^ We have further refined this sampling platform and demonstrated the ability to detect a wide variety of proteomic components in brain ISF with less than 1 μL of sample volume. We analyzed the proteomic components in ISF from two different brain regions, the substantia nigra (SN) and the nucleus accumbens (NA), and we compared these findings with the proteomics of CSF. We found significant difference in both protein abundance and type between fluid sampled from different brain regions (NA *vs.* SN) as well as compartments (ISF *vs.* CSF). Our findings show that ISF sampling may offer a more comprehensive liquid biopsy compared to CSF by capturing a broader range of proteins and molecular signatures. We also found that differences in protein expression in ISF from different regions could be connected to its biological function, emphasizing the need for regional specificity when sampling for clinical purposes. Our membrane-free approach, combined with the high spatial resolution of our sampling, provides a powerful tool for studying discrete brain regions and their molecular signatures, enabling more precise insights into neurobiological processes and disease mechanisms.

## Methods

### Device design, fabrication, and *in vitro* characterization

The device comprises a poly ether-ether-ketone (PEEK) body (1.59 mm outer diameter, 250 μm inner diameter, IDEX Health & Science) and a borosilicate capillary (80 μm outer diameter, 50 μm inner diameter, VitroCom Inc.). The capillary was inserted into the PEEK tubing and sealed with a biocompatible UV-curable adhesive (Loctite 4305, Henkel Corp). Capillary lengths of 8.2, 9.4, or 5 mm targeted the nucleus accumbens, substantia nigra, or lateral ventricle, respectively. Assembled devices were mounted in a custom 3D printed holder that aligned all capillaries to the same height. The holder was mounted in ULTRAPOL polisher (ULTRA TEC Manufacturing, Inc.) with a 45° tip angle. Tips were polished in batches for several hours and typically left overnight. Devices were then flushed with ethanol to remove polishing residue and clean the inner lumen. All devices were sterilized by autoclave before animal use.

Devices were tested in a brain phantom (300 mg agarose/50 mL water) for *in vitro* characterization to optimize recovered volume by adjusting infusion rate, withdrawal rate, and infused volume. The capillary was inserted 7 mm into the phantom, and Trypan Blue dye was used to visualize sampling. Dead volume was measured in the entire sampling system by infusing dye at 0.1 μL min^−1^ and measuring transit time at five points using the *in vivo* setup.

### Animals and surgical procedure

All animal protocols were approved by the Massachusetts Institute of Technology Committee for Animal Care. Female Sprague Dawley rats (Charles River Laboratories) were housed under a 12-hour light/dark cycle in an SPF facility at the Koch Institute, MIT, with *ad libitum* access to chow and water. All procedures followed the MIT IACUC-approved protocol (#2304000501). The animals had a mean weight of 268 ± 10 g (range: 257–290 g; *n* = 19).

Rats were anesthetized with isoflurane (1–4% in oxygen) and subcutaneous buprenorphine (1 mg kg^−1^), then secured in a stereotactic frame (Stoelting Co). Anesthesia depth was confirmed *via* pedal withdrawal reflex, and respiration was continuously monitored. Body temperature was maintained with heating pads. A ∼1.5 cm incision was made from the anterior skull to the posterior ridge, and burr holes were drilled using stereotaxic coordinates based on the Paxinos and Watson Rat Brain Atlas^72^ (nucleus accumbens: AP 1.5, ML ±1, DV −7.2; substantia nigra: AP −5, ML ±2.5, DV −8.4; lateral ventricle: AP −0.7, ML ±1.4, DV −4). Normal saline was applied to prevent tissue desiccation.

### BSA coating, sample collection, and contamination control

The pre-coated Hamilton syringe was directly coupled to the sampling device inlet without any intervening tubing to minimize protein adsorption. The collection tubes were also BSA-coated before sampling. 40 μL of 25 ng μL^−1^ digested BSA peptides were dried *via* speed vacuum, followed by two washes with 10% acetonitrile in 0.1% acetic acid and a final rinse with 0.1% acetic acid. Tubes were vortexed, centrifuged between washes, and stored at −80 °C until use.

The sampling device was mounted on a stereotaxic frame for sample collection, and the cannula was lowered into the target brain region. A 10 μL Hamilton syringe (32G needle), pre-coated with 1 pM digested BSA, was connected to the device. ISF and CSF were collected *via* a push-pull method: 2 μL of artificial CSF (aCSF) was infused at 0.3 μL min^−1^, followed by withdrawal at 0.2 μL min^−1^. Samples were stored in BSA-coated polypropylene tubes (1.5 mL, VWR) on solid carbon dioxide, then transferred to −80 °C. Rats were euthanized after sampling, and probe placement was verified visually and histologically. Samples with incorrect placement were discarded (NA: 3, SN: 2, ventricle: 4), with higher yield in later procedures.

The implantation site was thoroughly washed with saline before insertion to limit blood contamination, and sampling was delayed until bleeding subsided. A control sample (2 μL aCSF pushed through the device into a tube) was taken before *in vivo* collection to identify and exclude contamination-related proteins during data processing.

### *In vivo* pressure measurement and histological analysis

Gauge pressure at the probe inlet was monitored using a fiber optic pressure sensor (FISO FOP-LS-PT9-10). A custom tri-inlet adapter, laser-cut from clear acrylic, connected the probe, sensor, and syringe needle *via* perpendicular channels and SEBS tubing gaskets. The probe tip remained at atmospheric pressure, allowing measurement of pressure changes during fluid infusion/withdrawal *via* a syringe pump (Fig. S1).

Rats were euthanized after sampling, and 2 μL Trypan Blue was infused to trace the device trajectory. The brain was collected, fixed in 10% neutral buffered formalin, dehydrated, paraffin-embedded, sectioned perpendicular to the probe axis, and stained with H&E at 60 μm intervals.

### Micro-CT imaging for volume analysis

Sprague Dawley rats (*n* = 4) were implanted with sampling devices and imaged *via* micro-CT to assess infusion volume coverage. Bilateral craniotomy targeted the nucleus accumbens (AP 1.5, ML ±1, DV −7.2), and devices were cemented with dental acrylic. Incisions were sutured, and rats received 1 mg kg^−1^ buprenorphine and 0.9% saline postoperatively. Rats were anesthetized (3% isoflurane induction, 1–2% maintenance) one week later. Iohexol contrast (Omnipaque 350, 175 mg I per mL) was infused (2 μL at 0.3 μL min^−1^) using a Stereotaxic Injector (Stoelting Co.). Imaging was performed immediately with a Skyscan 1276 CMOS micro-CT scanner (Bruker) under 32-micron resolution with 0.4° rotation steps. Images were reconstructed (NRecon, Micro Photonics Inc.) and analyzed using Dragonfly (ORS, v2022.1). A 3D region of interest (ROI) was created using thresholding (edges at 60% peak intensity), and volume estimates were extracted.

### Mass spectrometry analysis and data processing

Protein samples were diluted (50 μL, 100 mM ammonium bicarbonate), reduced (10 mM dithiothreitol, 56 °C, 1 h), alkylated (20 mM iodoacetamide, 25 °C, dark, 1 h), and digested overnight with trypsin (Promega, 25 °C). Digestion was stopped with 5% formic acid, and peptides were desalted (Pierce Peptide Desalting Spin Columns, Thermo Fisher). Peptides were separated *via* HPLC (Thermo Ultimate 3000, PepMap RSLC C18, 2 μm, 75 μm × 50 cm, 60-min gradient), ionized, and analyzed on a Thermo Orbitrap Exploris 480 mass spectrometer in data-dependent acquisition mode. Full MS scans (375–1500 *m*/*z*) were performed at 60000 resolution, followed by 15 MS/MS scans (28 collision energy, 20 s dynamic exclusion, 30 000 resolution).

Raw data was analyzed using Proteome Discoverer 2.5 (Thermo Fisher) and Mascot 2.4.1 (Matrix Science) with 10 ppm precursor and 15 mmu fragment ion tolerances and up to 2 missed trypsin cleavages. Carbamidomethylation (Cys) was fixed, while methionine oxidation was variable. Only peptides with Mascot score ≥25 were considered. Data was searched against the *Rattus norvegicus* database (SwissProt), with single-instance proteins and keratin-type contaminants (Table S1) excluded. Missing values were mean-filled if present in >50% of replicates, otherwise excluded.^73^

### Data visualization and statistics

All data processing was done using R 4.3.0 (IDE RStudio RStudio 2023.03.0 + 386 “Cherry Blossom” Release) or R 4.4.1 (IDE RStudio 2024.04.2 + 764) and GraphPad Prism 9. Protein annotations were obtained from the GO database.^74,75^ The PCA and volcano scatter plots were created using ggplot2,^76^ and the correlation and abundance heatmaps were generated using Bioconductor package ComplexHeatmap.^77,78^ Pathway enrichment analysis was performed using the Bioconductor package clusterProfiler.^79–81^ The reactome pathway database and the molecular signatures database (MSigDB) database were used for analysis.^82–84^ Differences between groups were compared using an *F*-test to determine a difference in variance. This was followed by unpaired, two-tailed *t*-tests. A *P* ≤ 0.05 was considered significant.

## Results

### Device design and *in vitro* characterization of sampling parameters

**Fig. 1:**
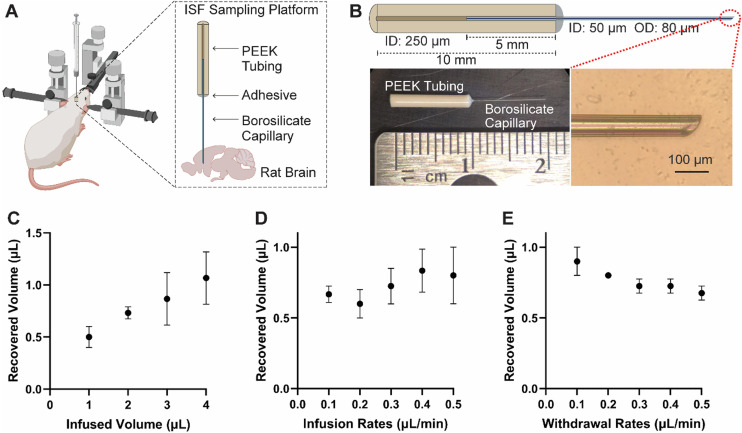
Design and *in vitro* characterization of micro-invasive sampling devices. (A) Schematic illustration of the device structure. (B) Optical images of the assembled device. (C–E) recovered volume with different (C) infused volume, (D) infusion rates and (E) withdrawal rates. For C–E, the infused volume was 2 μL, the infusion rate was 0.3 μL min^−1^, and the withdrawal rate was 0.2 μL min^−1^ for all tests where that parameter was not being evaluated (*N* = 3–5, values represent means ± SD). Analysis for C–E was done using one-way ANOVA.

We designed and developed a micro-invasive sampling device by interfacing polyether-ether-ketone (PEEK) tubing with polished borosilicate capillaries (Fig. 1A and B). We selected PEEK and borosilicate capillaries because PEEK offers biocompatibility, chemical stability, and a stiffness close to bone,^85^ while borosilicate glass is ideal for implantable devices due to its tiny size, chemical inertness, and ability to form reliable glass-to-metal seals.^86^ The PEEK tubing serves as the main structure, connecting the borosilicate capillary to commercially available micropumps. The tiny borosilicate capillaries are used as the implanted cannula and provide the ideal balance of stiffness and flexibility as shown in our previous studies.^70,71,87,88^ This allows for direct brain insertion without a guide tube and reduces the risk of breakage. Small lumens of this size demonstrate significantly less chronic glial scarring after 8 weeks as compared to larger diameter devices (≥150 μm).^70^ They also prevent the entry of large debris and do not clog.^88^ The polished tip of the capillary ensures smoother insertion and further minimizes tissue damage (Fig. 1B).

The small size of the borosilicate capillary significantly minimizes the internal volume of the device (dead volume) so that the PEEK body is the main contributor of the dead volume. Dead volume was determined by injecting a dye solution at a constant flow rate and recording the time it took to reach the capillary outlet (data not shown). This resulted in a measured dead volume of 0.16 ± 0.01 μL (*n* = 5), equivalent to approximately 3.5 mm of internal PEEK tubing volume.

We used a refined setup in this study compared to our previous work.^23^ Specifically, the Hamilton syringe was directly coupled to the sampling device, which reduced system dead volume and eliminated any connecting tubing. This design minimizes the internal surface area encountered by proteins during transport. Direct coupling reduces adsorption-related losses because proteins can adsorb to the tubing surfaces and thereby improves the accuracy of the measured ISF proteome.

Push–pull ISF sampling was performed using a programmable syringe pump (Stoelting Co.) coupled to a Hamilton syringe. The system infuses 2 μL of aCSF through the capillary and subsequently withdrew an equal volume (2 μL) through the same capillary. The withdrawn sample consisted of ISF mixed with the infused aCSF. The single-capillary design enables push–pull perfusion through a single lumen with a measured device dead volume of 0.16 μL and reduced wetted surface area, which is advantageous for low-loss proteomic sampling compared with push–pull implementations that require additional lumens and/or interconnect tubing.^51,52,55,58^

*In vitro* measurements with our push-pull sampling method were performed to evaluate the recovered volume across various parameters. Fig. 1C shows that as the total volume infused increases, the total volume recovered also increases (*P* ≤ 0.05). There is an upper limit to the amount of fluid that can be infused *in vivo*, however, as excessive fluid infusion can potentially damage brain tissue and limit spatial resolution. The total time required for sampling also increases proportionally with the infusion volume. We chose to use an infusion volume of 2 μL for our *in vivo* sampling to optimize the recovery volume while limiting the spatial distribution to just the brain region of interest.

Fig. 1D shows that no significant difference in recovery volume was observed with different infusion rates (*P* > 0.5). The rate of fluid withdrawal, by comparison, significantly affected the recovery volume with slower withdrawal rates leading to an increase in volume recovered (Fig. 1E) (*P* ≤ 0.05). We chose to infuse at a rate of 0.3 μL min^−1^ and to withdraw at a rate of 0.2 μL min^−1^ for our *in vivo* sampling experiments to maximize the recovered volume and minimize total sampling time. Each sample is collected over a 16 minute window under these settings. The collection window can be further shortened by increasing the flow rates. For example, using 0.5 μL min^−1^ for both infusion and withdrawal would reduce the collection window to 8 minutes for the same push–pull cycle. Compared with cerebral microdialysis used for brain proteomic analysis, our method uses a much shorter collection window. Brain microdialysis proteomics commonly collects hour-scale fractions (*e.g.*, 60–120 min or every 2 h), and samples may be pooled to obtain sufficient material.^89,90^ Seconds-scale temporal resolution has been demonstrated in push–pull perfusion for neurochemical monitoring, but those systems are primarily designed for small-molecule dynamics rather than low-loss protein/proteomics sampling.^55,63^ Our approach is membrane-free, which avoids protein losses and bias associated with adsorption to dialysis membranes. In addition, the shorter collection window reduces residence time in the fluidic path and can further limit adsorption-related losses before downstream processing.^91^

### *In vivo* characterization of sampling platform

**Fig. 2:**
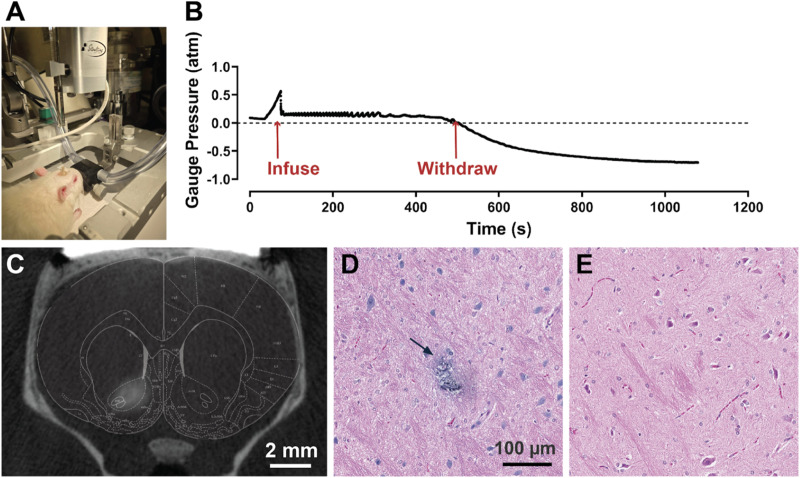
*In vivo* characterization of sampling platform. (A) Image showing sampling setup in an anesthetized rat. A 10 μL BSA-coated syringe is connected to an implanted device, and samples are taken using a push-pull method. (B) Gauge pressure measurements during the infusion and withdrawal process. (C) MicroCT image showing the affected area from the infused volume. (D) Representative hematoxylin and eosin (H&E) staining image showing implant radius (arrow). Trypan blue dye was infused to visualize the device trajectory resulting in the blue discoloration in the image. (E) Control histological image from the same brain location without device implantation. (*N* = 4).

The ISF sampling platform was characterized *in vivo* in anesthetized rats (Fig. 2A). Gauge pressure was measured during the sampling period using a pressure sensor positioned between the syringe needle and the device. Fig. 2B shows that gauge pressure increased at the start of the infusion as the aCSF displaced the surrounding tissue. The pressure stabilized once fluid flow became smooth, remaining slightly above atmospheric pressure. The gauge pressure dropped dramatically into the negative range when we began withdrawing the aCSF and ISF mixture. This shows why an initial infusion is necessary, as directly sampling ISF from the brain without it would be challenging due to the substantial pressure drop during withdrawal.

Micro-computed tomography (microCT) imaging following infusions of contrast agent were done *in vivo* to determine bolus delivery size and coverage. Imaging revealed the spatial distribution of a 2 μL infusion bolus was predominantly spherical and occupied a volume of 4.9 ± 0.25 mm^3^; demonstrating minimal backflow and diffusion away from the delivery site (Fig. 2C). This volume is considerably smaller than the nucleus accumbens, which is roughly 22 mm^3^ in rats, and equivalent to the size of the substantia nigra, which is about 5 mm^3^ in size.^92^ This shows that our sampling platform can achieve highly localized targeting and validates that we are sampling from the target region of interest.

Damage due to device insertion was also considered. Histological analysis of the implanted device's trajectory showed tissue damage of <80 μm, which corresponds to the outer diameter of the capillary (Fig. 2D). The capillary tip was polished to a smooth beveled profile, which reduces insertion forces and limits tearing at the tissue interface. This size is comparable to the shape and structure of the surrounding neurons.^93–95^ Furthermore, the implanted device elicited no discernible inflammatory response or necrotic tissue within the examined brain region compared to normal tissue in the same area (Fig. 2E). We have shown in previous studies that the use of micron-scale borosilicate capillaries with high bending stiffness and high aspect ratio minimizes neural tissue injury compared to stiffer, fixed cannulas.^70,71,87,88^ These findings combined with our previous data suggest that device implantation results in minimal tissue damage and disruption to the blood–brain barrier (BBB).

### Proteomic analysis of ISF and CSF samples

**Fig. 3:**
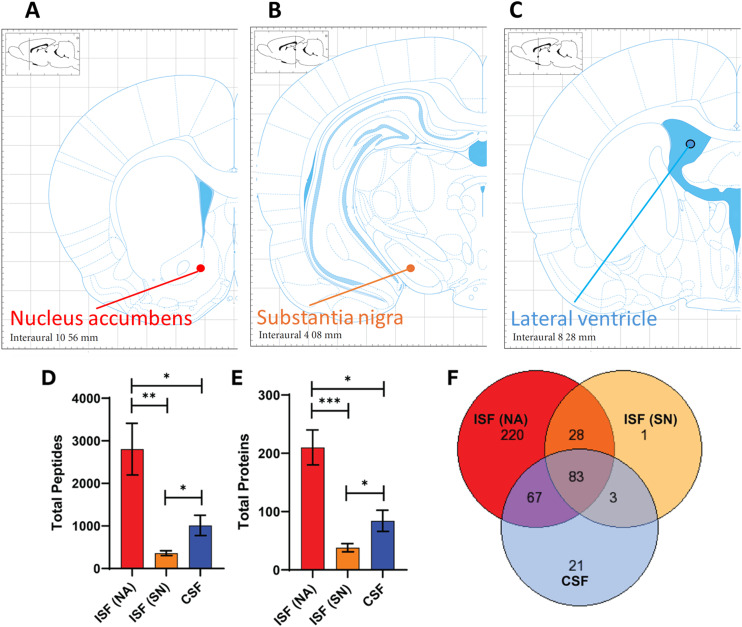
*In vivo* sampling of ISF and CSF in rat brain. (A–C) Brain locations for ISF and CSF sampling. ISF was sampled from the nucleus accumbens (NA) and substantia nigra (SN), and CSF was sampled from the lateral ventricle. Anatomic reference images courtesy of the Paxinos Rat Brain Atlas.^72^ (D) Total peptides and (E) total proteins identified by LC-MS/MS analysis. (F) Venn diagram showing the number of proteins identified in ISF (NA), ISF (SN), and CSF. For D and E, values represent mean ± standard error of the mean. Statistical differences between groups are indicated by asterisk (*p < 0.05; **p < 0.01, ***p < 0.001). *N* = 8 for ISF (NA), 6 for ISF (SN), 5 for CSF. (A–C) Were created/adapted using the online Rat Brain Atlas tool (Matt Gaidica; http://labs.gaidi.ca/rat-brain-atlas; accessed Jan 7, 2026), which is based on the Paxinos & Watson rat brain atlas.

We collected ISF samples from two brain regions, the nucleus accumbens (NA) and substantia nigra (SN), and CSF from the lateral ventricle (Fig. 3A–C). All samples were analyzed using LC-MS/MS with protocols optimized for proteomic analysis rather than small-molecule detection. Fig. 3D shows that the total number of peptides detected, which reflects protein abundance, was highest in ISF from the NA (mean = 2804 ± 1717), significantly exceeding levels in ISF taken from the SN (mean = 360 ± 139). A total of 571 unique proteins were detected across all ISF samples (*n* = 14). A total of 402 proteins were detected in the ISF after filtering out keratin-type proteins found in control samples taken during each procedure and proteins that appeared in only one sample. A significant portion (44%) of the proteins are extracellular or secretory proteins (Fig. S2). This is consistent with findings from other research.^96^Fig. 3E shows that the total number of proteins found in each ISF sample from the NA ranged from 93 to 324 (mean = 210 ± 85) while the number of proteins found in each ISF sample from the SN ranged from 15 to 59 (mean = 38 ± 17).

The total number of peptides detected in the CSF (mean = 1015 ± 539) was higher than ISF from the SN, but significantly lower than ISF from the NA (Fig. 3D). The number of unique proteins detected in the CSF after filtering was 174 (35–138 in each sample). This is lower compared to the ISF. When comparing CSF to the two ISF groups individually, however, CSF had significantly more proteins than ISF from the SN, but significantly less than ISF from the NA (Fig. 3E). The significant difference in both protein abundance and type between fluid sampled from different brain regions (NA *vs.* SN) as well as compartments (ISF *vs.* CSF) indicates the heterogeneity of different brain structures.

Out of the 423 proteins identified in both ISF and CSF, 83 (19.6%) were identified in all sample groups as shown in the Venn diagram (Fig. 3F). Some of the most abundant brain-specific proteins found in all groups were ATPase Na+/K+ transporting subunit alpha 3 (Atp1a3), heat shock protein family A (Hsp70) member 8 (Hspa8), and brain acid soluble protein 1 (Basp1). The majority of proteins identified (220; 52%) were found in ISF from the NA alone. The most abundant of these proteins were glutamate related proteins such as glutamine synthetase (Glul), as well as synaptic and vesicle related proteins such as vesicle-fusing ATPase (Nsf) and synaptotagmin-1 (Syt1). Only one protein, arginase-1 (Arg1), was isolated to just ISF from the SN. The largest overlap in proteins was between ISF from the NA and CSF, which had 67 proteins in common (15.8%). Many of these proteins were associated with enzymes involved in energy metabolism or binding activity, such as sodium/potassium-transporting ATPase and calcium/calmodulin-dependent protein kinase. The observation that CSF shared more proteins in common with ISF from the NA than ISF from the SN could be due to the proximity of the NA to the lateral ventricle. The next biggest overlap was between the two ISF groups, which shared 28 proteins in common (6.6%). Some brain-specific proteins of interest were growth associated protein 43 (Gap43), aldolase, fructose-bisphosphate C (Aldoc), and myelin-associated glycoprotein (mag). The complete list of proteins found in each group for the Venn diagram can be found in the SI.

**Fig. 4:**
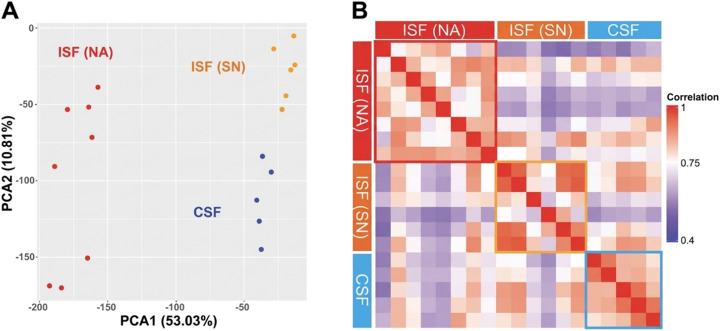
Comparative analysis of protein composition and correlation across brain regions (A) principal component analysis showing the major variance in protein composition between different brain regions can be explained by two principal components. (B) Pearson correlation heatmap displaying the matrix of similarities in protein profiles across different brain regions. *N* = 8 for ISF (NA), 6 for ISF (SN), 5 for CSF.

We assessed the sensitivity and reproducibility of our sampling method by comparing individual sample profiles from each group using principal component analysis (PCA) and generating a Pearson correlation heatmap. The PCA in Fig. 4A revealed that over 60% of the variance among the groups could be explained by two principal components. This suggests that the differences between the groups are significant and can be captured effectively using only two key variables. The ISF samples showed a wider spread along PCA2. We do not interpret this as clear evidence of subgroups. In mass spectrometry proteomics, a protein can be present in a sample but still not be detected in every run, especially in small-volume samples and for low-abundance proteins. This can lead to missing values and make samples look more spread out in PCA. The correlation heatmap in Fig. 4B shows that samples from the same group were highly correlated, while samples from different groups had low correlation. The strong correlations within groups and the significant variance explained by the PCA components suggest that the sampling method used is both sensitive and reproducible, providing reliable and distinct data across different brain regions.

### Proteomic comparison of ISF and CSF

**Fig. 5:**
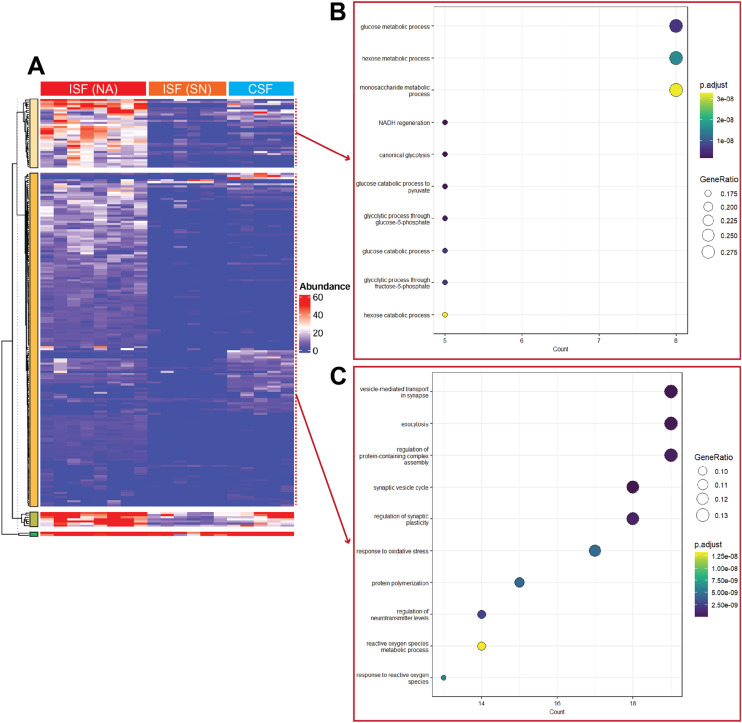
Proteomic analysis of the brain ISF and CSF. (A) Hierarchical clustering of proteomic profiles from ISF (NA), ISF (SN), and CSF samples. (B) and (C) Pathway enrichment analysis of individual protein clusters. *N* = 8 for ISF (NA), 6 for ISF (SN), 5 for CSF.

We aimed to investigate the biological differences between ISF and CSF, as well as variations in ISF composition across different locations. We hypothesized that fluid from different brain regions and compartments would differ in their patterns of protein expression. Our analysis revealed that brain ISF in the NA region contains significantly more proteins compared to ISF in the SN region and CSF. Hierarchical clustering of the protein data identified distinct groups among the ISF (NA), ISF (SN), and CSF samples (Fig. 5A). This result is consistent with the correlation heatmap in Fig. 4B and supports the conclusion that samples were more similar within groups than across groups, even though the ISF samples showed a wider spread in the PCA plot. We also conducted pathway enrichment analysis to examine the biological pathways associated with protein expression. Fig. 5B shows the pathway enrichment for the second-largest cluster, indicating that the proteins in this group are involved in metabolic processes, with an emphasis on glucose-related processes such as glucolysis/gluconeogenesis. Fig. 5C presents the pathway enrichment for the largest cluster, whose proteins are involved in synaptic transmission, regulation, and vesicle-mediated transport. The proteomic composition of ISF from the NA suggests a higher level of metabolic activity and neurotransmission than the SN. This finding is consistent with research demonstrating that the striatum (where NA is located) has a higher synaptic density than the midbrain (where SN is located).^97^ The elevated release of extracellular vesicles may indicate increased intercellular communication and synaptic plasticity, which are critical for modulating neural activity in processes like learning, memory, and reward.^98^ Additionally, the broader range of metabolic activities observed in the NA may reflect the region's energy demands, as synaptic transmission and plasticity require significant metabolic resources to sustain neurotransmitter cycling, ion channel activity, and vesicle recycling.^99^

**Fig. 6:**
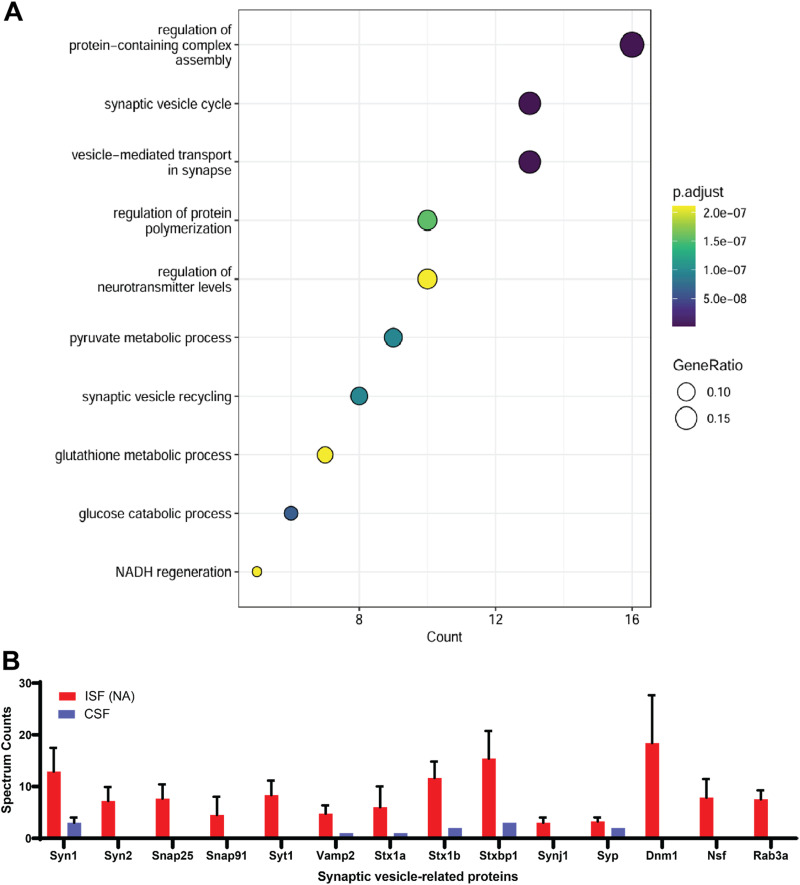
Differential protein expression and pathway enrichment between brain ISF and CSF proteomes. (A) Pathway enrichment analysis comparing proteins uniquely found in ISF (NA) with those significantly more abundant in ISF (NA) than in CSF. (B) Bar graph illustrating the abundance of important synaptic vesicle-related proteins in ISF (NA) compared to CSF. Data values are shown as mean ± SEM.

We compared the proteomic compositions of ISF (NA) with CSF to identify pathways associated with differentially expressed proteins. Common proteins identified in both CSF and ISF (NA) proteomes were analyzed to generate a volcano plot (Fig. S3). Of the 24 proteins that exceeded the *p* < 0.05 significance threshold, 22 were significantly more abundant in ISF (NA) compared to CSF, while only two were more abundant in CSF. We conducted a pathway enrichment analysis by combining the proteins uniquely detected in ISF (NA) with those significantly more abundant in this region to explore the biological processes associated with these differentially expressed proteins in the NA. Fig. 6A shows the pathways where ISF (NA) diverges from CSF, highlighting key processes such as the synaptic vesicle cycle and various metabolic pathways.

Fig. 6B highlights key synaptic vesicle-related proteins identified in ISF (NA). Included in this group are Synaptosomal-Associated Protein 25 (Snap25), Vesicle-Associated Membrane Protein 2 (Vamp2), syntaxin 1A/1B (Stx1a and Stx1b), and syntaxin-binding protein 1 (Stxbp1), all of which are SNARE (Soluble NSF Attachment Protein Receptor) proteins or closely related to them. This family is essential for membrane fusion and the release of neurotransmitters into the synaptic cleft.^100,101^ Other proteins identified include synapsin 1 (Syn1), synapsin 2 (Syn2), synaptophysin (Syp), synaptojanin 1 (Synj1), and synaptotagmin 1 (Syt1), which are involved in vesicle fusion, trafficking, and recycling.^102,103^ Additionally, dynamin 1 (Dnm1) and Rab3a are GTPases that play important roles in vesicle scission and the regulation of exocytosis.^104,105^ These findings indicate that ISF sampling may offer a more comprehensive liquid biopsy compared to CSF by capturing a broader range of proteins and molecular signatures that are more specific to brain activity and pathology.

## Discussion

A comprehensive understanding of the CSF and ISF proteomes and their biological linkage is essential for investigating pathological changes in the brain. Previous studies have performed proteomic analyses of both CSF and ISF fluid compartments using intracerebral microdialysis combined with proteomic LC-MS/MS analysis. Our group previously introduced a micro-invasive platform for brain ISF sampling and demonstrated proof-of-concept detection of extracellular proteins by mass spectrometry, where a nitinol-activated nanofluidic pump was used for fluid collection.^23^ And in this work, we improved the platform by directly interfacing the sampling syringe, thereby reducing dead volume and increasing protein recovery efficiency. We were able to detect a broad range of ISF proteomic components from less than 1 μL of sample with this refinement. In our previous work a total of 120 proteins were detected in the substantia nigra (*n* = 3),^23^ while here we identified 115 proteins (*n* = 6) in the same region, with 48 proteins overlapping between the two datasets. Although the number of identified proteins in SN is similar between studies, the current work achieves comparable identification with smaller collected volumes (<1 μL) and a stricter contamination-control workflow by excluding proteins detected in controls. Also, this study increases biological replication and extends sampling across multiple regions/CSF, enabling pathway-level comparisons not addressed in previous work.

In another work, Bernay *et al.*^96^ identified 88 proteins in the rat striatum using microdialysis probes with molecular weight cut-offs of 20 and 100 kDa. Our work sampled from nucleus accumbens, which is a sub brain region of the striatum, where we detected 398 proteins, 70 of which overlapped with those reported by Bernay *et al.* This overlap accounts for most of the proteins identified in their work. We identified more proteins because we used membrane-free sampling platform, which avoids the size exclusion inherent to microdialysis probes and thus allows recovery of larger proteins. Overall, our proteomic findings are consistent with prior reports while also demonstrating the advantages of our method. Compared with microdialysis, which requires extended sampling times and is less spatially precise, our approach enables localized ISF sampling from a single brain region within a shorter timeframe.

Our study sought to overcome these challenges by demonstrating micro-invasive, membrane-free, rapid sampling of ISF fluid for proteomic analysis. Samples were collected in only 16 minutes with each sample containing less than 1 μL of ISF. A total of 571 unique proteins were detected in ISF and 206 proteins in CSF before data was manually filtered. After filtering data to exclude proteins appearing in less than two samples and keratin-like proteins related to contamination during sample collection, a total of 402 unique proteins were identified in the ISF and 174 were identified in the CSF with molecular weights ranging from 5 kDa to 332 kDa. The average number of unique proteins detected in a single ISF sample (<1 μL) from the NA was 223, which highlights the platform's capacity to yield a rich proteomic profile from minute sample sizes. Even after filtering, these numbers are comparable to or even greater than the microdialysis studies mentioned above which did not employ our strict filtering criteria, and whose sample time (>1 hour) and volume (>30 μL per sample) were significantly larger than ours.

We also compared our ISF proteomic profiles with published brain tissue proteomes from the same regions. We identified 398 unique proteins in the nucleus accumbens (NA) within the striatum (*n* = 8), whereas a prior tissue study of synaptosomes reported 3431 proteins, with 242 proteins overlapping between datasets (*n* = 3).^106^ We identified 115 proteins in the substantia nigra (SN) within midbrain (*n* = 6), whereas a prior tissue proteome from the same region reported 2985 proteins (*n* = 4, different age groups), with 91 proteins overlapping.^107^ The overlap reflects proteins that exist in both intracellular and extracellular compartments. Our membrane-free platform also samples extracellular vesicles, enabling detection of vesicle-associated surface proteins and proteins involved in exocytosis, which also contributes to shared identifications across ISF and tissue measurements. We show through our *in vivo* imaging study that the region of brain tissue we are sampling from is roughly 4.9 mm^3^. This allows us to sample with high specificity from discrete brain microstructures such as the nucleus accumbens core and the substantia nigra in, which are roughly 21.8 mm^3^ and 4.7 mm^3^ in size respectively.^92^ This high level of spatial resolution would not be possible with microdialysis techniques which are limited by their relatively large exchange membrane area (diameter of 400 μm, length of 1–4 mm).^37,61,108^ Our membrane-less design also provides an advantage over microdialysis. We have shown in a previous *in vitro* study that our sampling platform significantly outperformed microdialysis in its ability to measure larger molecules such as myelin basic protein (MBP; ∼13 to 21 kDa) and hemoglobin (64 458 Da).^23^ It also demonstrated minimal extraction loss of MBP compared to microdialysis. These findings highlight the potential of our sampling platform as a superior tool for ISF sampling in clinical settings.

One of the goals of this study was to present the ISF as a viable and complementary source for the identification, validation, and characterization of brain biomarkers for diagnosing and monitoring neurological disease states. We found that the composition of normal CSF differs markedly from the composition of intracellular fluids in the brain. The overlap in proteins detected in both ISF and CSF (36%) clearly shows the physiological connection between these two brain fluids, but the large number of proteins identified in only the ISF compared to the CSF (249 *vs.* 21) emphasizes the relevance of the ISF as an important reservoir for the validation and discovery of new biomarkers. We further showed in our volcano plot in Fig. S3, that of the proteins common to both the ISF and CSF, all but two were significantly more abundant in ISF compared to CSF. Many of these proteins were synaptic vesicle-related proteins. We also identified transmembrane proteins carried by extracellular vesicles. Extracellular vesicles are especially valuable for diagnosing and detecting brain diseases because they transport an array of disease-specific proteins that can serve as biomarkers.^109^ Extracellular vesicles released from neurons, astrocytes, and other brain cells contain proteins that indicate pathological changes in the brain in neurological disorders such as Alzheimer's disease, Parkinson's disease, and multiple sclerosis.^110,111^ The contents of extracellular vesicles often get internalized and cleared by local cells and would not be detected in CSF samples.^112,113^ This suggests that ISF could provide novel insights into brain pathology that CSF may not capture.

CSF sampling is still a valuable diagnostic tool, particularly for diseases where established biomarkers are already known. The two proteins more abundant in CSF were cystatin 3 (Cst3), a peptidase inhibitor with brain-biased expression, and apolipoprotein E (Apoe), a protein component of lipoprotein particles in plasma and CSF. The Apoe gene is one of the strongest risk factors identified in Alzheimer's disease, and levels of apolipoprotein E in CSF fluid have been associated with biomarkers of Alzheimer's disease.^114–116^ Their greater abundance in CSF indicates that while ISF may have unique advantages, CSF still harbors essential biomarkers crucial for understanding and diagnosing certain neurological diseases. Utilizing both ISF and CSF could provide a more comprehensive understanding of neurological diseases.

We also demonstrated that this method is sensitive to the functional differences between brain regions. Specifically, sampling ISF from two different brain microstructures, the nucleus accumbens and the substantia nigra, show significant differences in protein abundance and composition indicative of their respective functions. We observed higher levels of protein expression in the NA than the SN, as mentioned in the results. This is consistent with previous work showing higher levels of protein content in the striatum compared to the midbrain in both human and rat brains.^117^ Most of the proteins found in the NA were associated with metabolic processes or synaptic transmission which also agrees with research demonstrating that the striatum has a higher synaptic density than the midbrain.^97,118^ This heightened activity in the NA region could be attributed to its role within the brain's reward and motivation circuits, which are known for their high synaptic density and dynamic neuronal signaling.^119,120^ The substantia nigra by comparison, is primarily involved in motor control and dopamine regulation, which are more basal neurophysiological functions.^121^ Pathway enrichment analysis of proteins detected in the SN (Fig. S4) were more frequently linked to cellular processes such as biosynthesis and metabolism, not synaptic transmission or regulation. Several proteins expressed in the SN are linked to Parkinson's disease such as heat shock protein family A (Hsp70) member 8 (Hspa8) and 14-3-3 proteins.^122,123^ These region-specific proteomic profiles underscore the importance of sampling ISF from distinct brain areas to gain insights into localized biochemical activity, potentially aiding in the development of targeted biomarkers for neurological diseases affecting different brain regions.

The results of our study demonstrate the potential of our sampling platform as a tool for diagnosing disease onset and monitoring disease progression through micro-invasive sampling of brain ISF. The ability to detect hundreds of proteins in a minute volume of ISF from a specific brain region offers several advantages: it allows for high-resolution, region-specific analysis of brain proteomics, enables more frequent sampling without causing significant damage or inflammation, and makes the technique more clinically viable. Furthermore, the detection of such a diverse range of proteins, unrestricted by molecular weight and size, provides valuable insights which would not be possible with current microdialysis techniques.

## Conflicts of interest

The authors declare that they have no competing interests.

## Supplementary Material

LC-026-D6LC00038J-s001

LC-026-D6LC00038J-s002

## Data Availability

The processed proteomics data supporting the findings of this study are provided in the supplementary information (SI). Details of data processing and analysis procedures are described in the Methods section of the manuscript. Supplementary information is available. See DOI: https://doi.org/10.1039/d6lc00038j.
