# Supplementary material for: Region-specific proteomic profiling of brain interstitial fluid via a micro-invasive sampling platform
Source: Lab Chip. 2026 Apr 13;26(10):3153–67. doi: 10.1039/d6lc00038j (PMC13096881; doi:10.1039/d6lc00038j)
Supplement: LC-026-D6LC00038J-s002 [file LC-026-D6LC00038J-s002.pdf]

This journal is © The Royal Society of Chemistry 2026

List of Supplementary Materials

Fig S1 Gauge pressure measurement setup.
Fig S2 Grouping of identified proteins by cellular localization.
Fig S3 Volcano plot showing common proteins that are differentially expressed between ISF from the nucleus accumbens (NA) and CSF.
Fig S4 Pathway enrichment analysis of proteins detected in the substantia nigra.
Table S1 List of proteins identified in control samples.

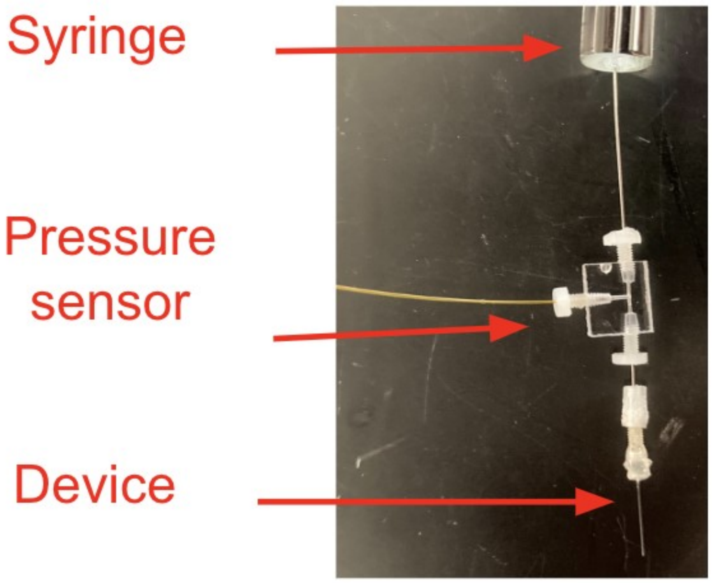

Figure S1 Gauge pressure measurement setup. The inlet pressure of the probe was monitored using a fiber optic pressure sensor (FISO Technologies, FOP-LS-PT9-10). A specially designed tri-inlet adapter facilitated the connection between the probe, sensor, and syringe needle. The probe tip was maintained at atmospheric pressure, enabling the measurement of pressure variations across the probe during fluid infusion or withdrawal by a syringe pump.

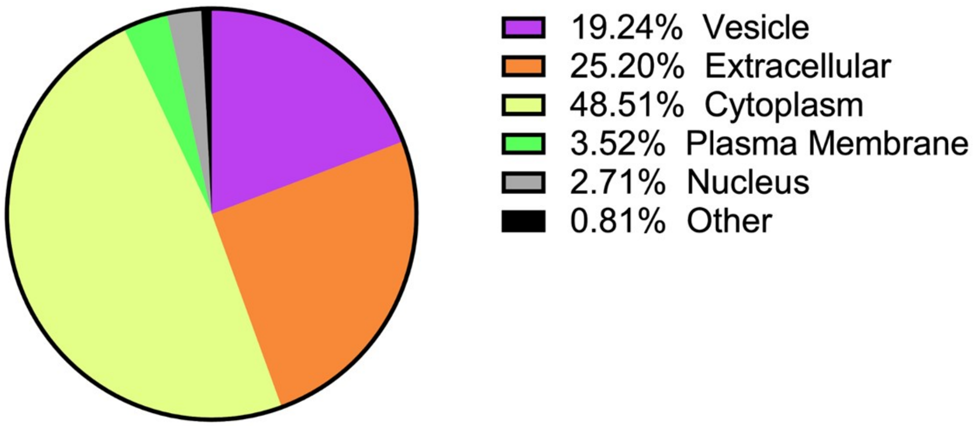

Figure S2 Grouping of identified proteins by cellular localization. Cellular localization data was obtained from the GO database.

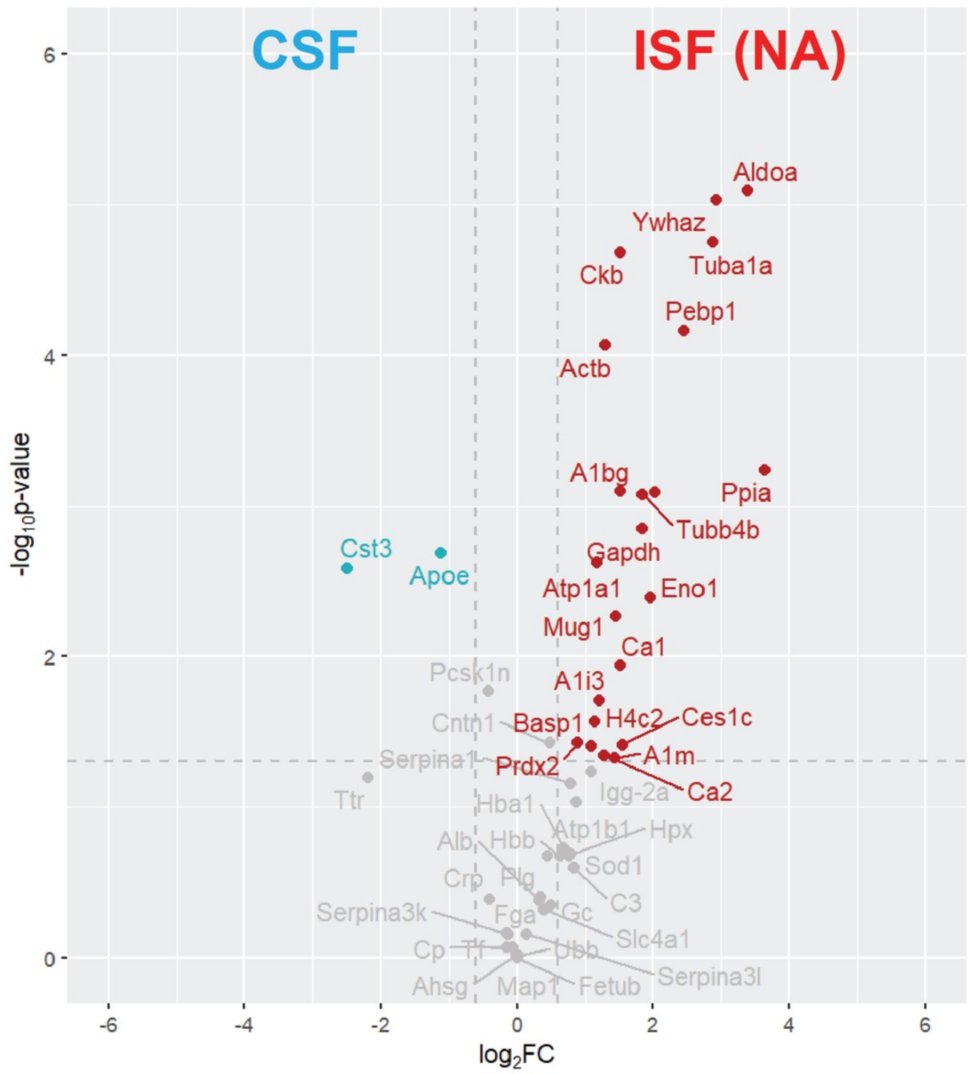

Figure S3 Volcano plot showing common proteins that are differentially expressed between ISF from the nucleus accumbens (NA) and CSF. The horizontal dotted line represents a significance level of $p = 0.05$ ($–\log_{10} = 1.3$), and the vertical dotted lines represent a fold change of ± 1.5. Only proteins found in both ISF (NA) and CSF were included in the plot. Proteins found in both groups but lacking statistical significance—such as those detected in only a single animal within a group—were excluded.

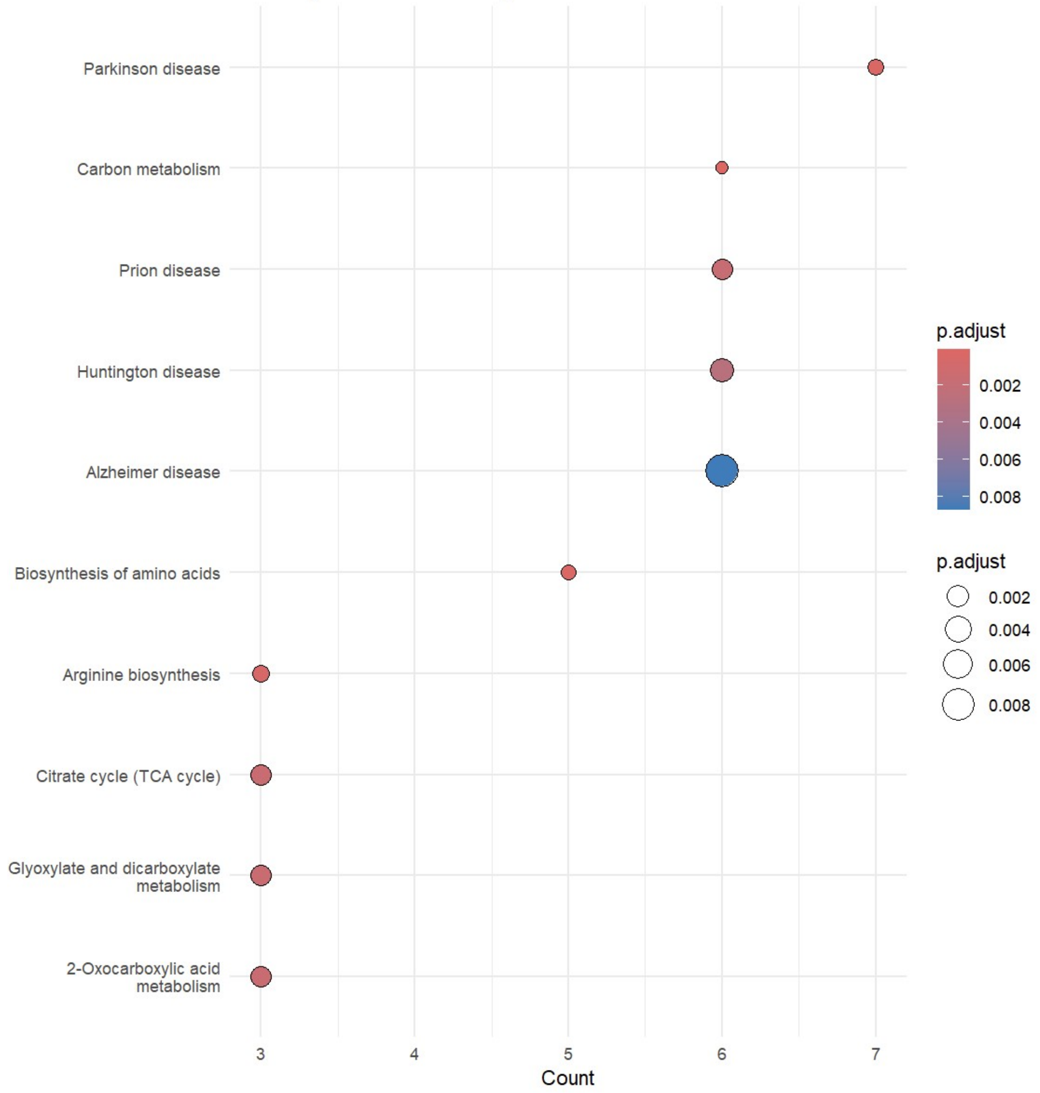

Figure S4 Pathway enrichment analysis of proteins detected in the substantia nigra. Pathway enrichment analysis was performed using the Bioconductor package clusterProfiler.

Table S1 List of proteins identified in control samples.

Proteins Identified	Accession Number	Alternate ID	Molecular Weight
Cationic trypsin-3 OS=Rattus norvegicus OX=10116 GN=Try3 PE=2 SV=1	P08426	Try3	26 kDa
Desmoplakin OS=Rattus norvegicus OX=10116 GN=Dsp PE=1 SV=1	F1LMV6	Dsp	332 kDa
Histone H4 OS=Rattus norvegicus OX=10116 GN=H4c2 PE=1 SV=2	P62804	H4c2	11 kDa
Junction plakoglobin OS=Rattus norvegicus OX=10116 GN=Jup PE=1 SV=1	Q6P0K8	Jup	82 kDa
Keratin, type I cytoskeletal 10 OS=Rattus norvegicus OX=10116 GN=Krt10 PE=3 SV=1	Q6IFW6	Krt10	57 kDa
Keratin, type I cytoskeletal 14 OS=Rattus norvegicus OX=10116 GN=Krt14 PE=2 SV=1	Q6IFV1	Krt14	53 kDa
Keratin, type I cytoskeletal 17 OS=Rattus norvegicus OX=10116 GN=Krt17 PE=1 SV=1	Q6IFU8	Krt17	48 kDa
Keratin, type I cytoskeletal 18 OS=Rattus norvegicus OX=10116 GN=Krt18 PE=1 SV=3	Q5BJY9	Krt18	48 kDa
Keratin, type I cytoskeletal 19 OS=Rattus norvegicus OX=10116 GN=Krt19 PE=1 SV=2	Q63279	Krt19	45 kDa
Keratin, type I cytoskeletal 42 OS=Rattus norvegicus OX=10116 GN=Krt42 PE=3 SV=1	Q6IFU7	Krt42	50 kDa
Keratin, type II cytoskeletal 1 OS=Rattus norvegicus OX=10116 GN=Krt1 PE=2 SV=1	Q6IMF3	Krt1	65 kDa
Keratin, type II cytoskeletal 2 epidermal OS=Rattus norvegicus OX=10116 GN=Krt2 PE=3 SV=1	Q6IG02	Krt2	69 kDa
Keratin, type II cytoskeletal 5 OS=Rattus norvegicus OX=10116 GN=Krt5 PE=1 SV=1	Q6P6Q2	Krt5	62 kDa
Keratin, type II cytoskeletal 6A OS=Rattus norvegicus OX=10116 GN=Krt6a PE=1 SV=1	Q4FZU2	Krt6a	59 kDa
Keratin, type II cytoskeletal 72 OS=Rattus norvegicus OX=10116 GN=Krt72 PE=3 SV=2	Q6IG04	Krt72	57 kDa
Keratin, type II cytoskeletal 73 OS=Rattus norvegicus OX=10116 GN=Krt73 PE=1 SV=1	Q6IG03	Krt73	60 kDa
Keratin, type II cytoskeletal 75 OS=Rattus norvegicus OX=10116 GN=Krt75 PE=3 SV=2	Q6IG05	Krt75	59 kDa
Keratin, type II cytoskeletal 8 OS=Rattus norvegicus OX=10116 GN=Krt8 PE=1 SV=3	Q10758	Krt8	54 kDa
Keratin, type II cytoskeletal 80 OS=Rattus norvegicus OX=10116 GN=Krt80 PE=3 SV=1	Q6IMF1	Krt80	51 kDa
Polyubiquitin-B OS=Rattus norvegicus OX=10116 GN=Ubb PE=1 SV=1	P0CG51	Ubb	34 kDa
Pyruvate carboxylase, mitochondrial OS=Rattus norvegicus OX=10116 GN=Pc PE=1 SV=2	P52873	Pc	130 kDa
Serine protease 1 OS=Rattus norvegicus OX=10116 GN=Prss1 PE=1 SV=1	P00762	Prss1	26 kDa
